# Assessment of Non-Routine Events and Significant Physiological Disturbances during Emergency Department Evaluation after Pediatric Head Trauma

**DOI:** 10.1089/neur.2020.0043

**Published:** 2021-01-29

**Authors:** Emily C. Alberto, Allison R. Harvey, Michael J. Amberson, Yinan Zheng, Arunachalam A. Thenappan, Chima Oluigbo, Ivan Marsic, Aleksandra Sarcevic, Karen J. O'Connell, Randall S. Burd

**Affiliations:** ^1^ Division of Trauma and Burn Surgery, Children's National Hospital, Washington DC, USA.; ^2^ Department of Pediatrics, Children's National Hospital, Washington DC, USA.; ^3^ Division of Neurosurgery, Children's National Hospital, Washington DC, USA.; ^4^ Department of Electrical and Computer Engineering, Rutgers University, Piscataway, New Jersey, USA.; ^5^ College of Computing and Informatics, Drexel University, Philadelphia, Pennsylvania, USA.; ^6^ Division of Emergency Medicine, Children's National Hospital, Washington DC, USA.

**Keywords:** brain injuries, errors, medical, pediatrics, protocol compliance, traumatic, vital signs

## Abstract

Outcomes following pediatric traumatic brain injury (TBI) are dependent on initial injury severity and prevention of secondary injury. Hypoxia, hypotension, and hyperventilation following TBI are associated with increased mortality. The purpose of this study was to determine the association of non-routine events (NREs) during the initial resuscitation phase with these physiological disturbances. We conducted a video review of pediatric trauma resuscitations of patients with suspected TBI and Glasgow Coma Scale (GCS) scores <13. NREs were rated as “momentary” if task progression was delayed by <1 min and “moderate” if delayed by >1 min. Vital sign monitor data were used to identify periods of significant physiological disturbances. We calculated the association between the rate of overall and moderate NREs per case and the proportion of cases with abnormal vital signs using multi-variate linear regression, controlling for GCS score and need for intubation. Among 26 resuscitations, 604 NREs were identified with a median of 23 (interquartile range [IQR] 17–27.8, range 5–44) per case. Moderate delay NREs occurred in 19 resuscitations (*n* = 32, median 1 NRE/resuscitation, IQR 0.3–1, range 0–5). Oxygen desaturation and respiratory depression were associated with a greater rate of moderate NREs (*p* = 0.008, *p* < 0.001, respectively). We observed no association between duration of hypotension, desaturation, and respiratory depression and overall NRE rate. NREs are common in the initial resuscitation of children with moderate to severe TBI. Episodes of hypoxia and respiratory depression are associated with NREs that cause a moderate delay in task progression. Conformance with resuscitation guidelines is needed to prevent physiological events associated with adverse outcomes following pediatric TBI.

## Introduction

Traumatic brain injury (TBI) is a leading cause of morbidity and mortality in children, affecting an estimated 475,000 children each year.^1–3^ Children with moderate to severe TBI (Glasgow coma scale [GCS] score <13) require immediate management to prevent or mitigate secondary brain injury. Guidelines have been developed for standardizing care during the early resuscitation phase, with a particular focus on prevention of hypotension, hypoxia, and hypo/hypercarbia.^4–6^ Adherence to established guidelines in the early resuscitative phase improves outcomes among children with TBI, with a 1% increase in adherence associated with a 6% decrease in mortality.^7^ Despite the strong evidence of its benefits, guideline adherence for children with TBI is variable, especially during the fast-paced, initial resuscitation phase of care.^8,9^ Both intentional non-adherence and unintentional errors contribute to deviation from established TBI protocols. Because of the risk of protocol deviation in the early resuscitation setting, a critical need exists to identify factors contributing to errors in TBI protocol adherence and the team's response to errors.^4^

In this study, we conducted a video review to identify and classify deviations from protocol during the initial resuscitation of children with TBI using non-routine event (NRE) analysis. NREs are events that are atypical or outside of what is expected. Identification of NREs has advantages over other methods of adverse event identification because it includes all types of atypical events, not only those linked with poor outcomes. This broad definition of what constitutes an NRE increases the number of observations and reduces observer bias that occurs with a focus on only events associated with a known adverse outcome.^10–12^ Identifying NREs and understanding their root cause are necessary for improving protocol adherence and team dynamics. The goal of this retrospective video review was to document the incidence of NREs, identify the most frequent types and causes of NREs, and identify the prevalence of significant physiological disturbances during resuscitation for pediatric TBI. Our long-term goal is to identify aspects of pediatric TBI resuscitation that can be improved through the understanding and elimination of NREs.

## Methods

### Setting

Children's National Hospital is an accredited Level 1 pediatric trauma center serving the greater Washington, DC area. About 600 injured children are managed annually as trauma activations based on institutional and regional triage criteria. Among the patients treated as trauma activations, about 40% present with mild (GCS score >12) to severe (GCS score <9) TBI. Patients are triaged to a three-tiered activation system with the highest-acuity patients triaged to the highest level (“attending”). Lower-acuity patients are triaged to two lower activation categories based on whether they are arriving from the scene (“stat”) or from another hospital (“transfer”).

The team evaluating trauma activations is multi-disciplinary, with team leadership shared between a surgical coordinator (pediatric surgery fellow or senior resident), an emergency department (ED) attending, and a documenting nurse. Other team members include a bedside surgical surveyor (junior resident or nurse practitioner) responsible for the physical assessment, one or more bedside nurses, and an airway team comprising an anesthesiologist and a respiratory therapist. A surgical attending and a critical care physician are immediately present for children triaged to the highest-level activation. For children with serious head injuries, a member of the neurosurgery team (resident, physician assistant, or attending neurosurgeon) is called to evaluate the patient within 30 min of arrival. Trauma activations are video recorded with views from overhead and the foot of the bed. Consent is required for video use for research purposes. The Children's National Hospital Institutional Review Board approved this study.

### Selection of participants

We conducted a retrospective, observational study of children (<15 years old) with a known or suspected TBI and an initial GCS score of <13 who were treated during a trauma activation between March 2018 and May 2019. We excluded patients with a GCS score <13 who did not have a head injury (e.g., drowning, thoracic/abdominal injuries with associated hemorrhagic shock). Sixty-three patients met the initial inclusion criteria. Twenty-two patients were excluded due to absent or poor-quality video and 15 because consent was not obtained for video review. Twenty-six patients were included in the final cohort.

### Study design

We obtained patient demographics, pre-arrival notification, mechanism of injury, injury severity score (ISS), and final ED and hospital disposition from the trauma registry. Medical record review and video review were used to obtain the time of the day, day of the week, the initial GCS score, and the need for intubation before arrival or in the trauma bay. Vital signs were sampled from the monitor every 30 sec and used to calculate the percent of the resuscitation with significant physiological disturbances: hypotension, hypertension, bradycardia, tachycardia, respiratory depression, and tachypnea. The significant physiological disturbances were defined as vital signs that were more than two standard deviations (SDs) outside age-appropriate norms or an oxygen desaturation below 92% (hypoxia).^13–15^ We separately considered the percent of the resuscitation with hypotension, hypoxia, and respiratory depression in the analyses because these abnormalities are independently associated with poor outcome after traumatic brain injury.^5,16–19^

Resuscitations were further subdivided into phases based on the Advanced Trauma Life Support (ATLS) protocol.^20^ The “pre-arrival” phase was defined as the time between team assembly and the patient's arrival. The “before primary survey” phase was defined as the time after patient arrival and before the first primary survey task. The “primary survey” and “secondary survey” phases were based on team activities, with the primary survey phase ending when the first secondary survey action begins, and the secondary survey phase ending at the completion of the log roll and back examination. The “post-secondary” phase was defined as the time between the end of the log roll and patient departure from the resuscitation room.

We modified a previously used data dictionary for NREs that was developed based on the ATLS protocol and expert opinion.^20,21^ Additions to the data dictionary for this study included NREs specific to neurotrauma, including “delay in 3% saline or mannitol administration” and “neurosurgeon late or absent.” Two ATLS-trained pediatric trauma surgeons and one Pediatric Advanced Life Support (PALS)-trained emergency medicine physician reviewed the data dictionary, assigning NRE “type” based on the features of the event. “Process events” were related to the action or inaction of the assembled trauma team and “non-process events” were defined as actions outside the team's control (e.g., equipment malfunction, parental actions). Process events were further subdivided into “errors of commission” when an incorrect or inappropriate action was taken to address a goal, “errors of omission” when the appropriate action was not taken, and “errors of selection” when the ordering of actions taken to address a goal were suboptimal.^21–23^

Three observers (one medical student and two resident physicians) were trained in NRE identification using videos of resuscitations not included in this study. The observers continued training until a consensus of at least 70% was achieved between reviewers. Following the training period, two observers independently reviewed each video to verify the results. When disagreements in coding occurred, the relevant section of the video was jointly watched by the observers to determine the final NRE assignment.

During individual coding, each observer assigned the “impact” of each NRE using two methods: the time expense to the team member and the potential for patient harm. “No delay” was assigned to NREs that were either unrecognized and not addressed or did not slow a provider's care (e.g., inadequate personal protective equipment use by providers), “momentary” was used for delays shorter than 1 min, and “moderate” was used for delays longer than 1 min.^23–25^ When assessing the potential for patient harm from the NRE, events were assigned as “major” when the potential for harm was high (e.g., failure to stabilize cervical spine) and “minor” when the potential was low (e.g., inadequate personal protective equipment use by providers).^23,26^ An NRE “cause” assessment was conducted to identify the factor or agent that lead to each event.^23^ To address the cause of each NRE, the observers identified the team member most associated with the event. Provider roles with similar tasks were grouped, with the anesthesiologist and respiratory therapist denoted as the “airway team.” The “leadership” role was assigned to NREs attributed to the surgical attending, surgical coordinator, ED attending, and documenting nurse. The “bedside team” included bedside nurses, the surgical surveyor, and the airway team who had direct interaction with the patient.

### Statistical analysis

Variables were described using median and interquartile ranges because of the non-normal distribution of NREs, resuscitation duration, and the percent of the resuscitation with significant physiological disturbances. To standardize the number of NREs among resuscitations of varying length, we divided the number of NREs per resuscitation by the length of time from patient arrival until patient departure. The length of each significant physiological disturbance was evaluated as a percentage of total resuscitation duration from patient arrival to departure. We calculated the associations between the rate of overall and major NREs per resuscitation and the proportions of each resuscitation with hypoxia, hypotension, or respiratory depression using multi-variate linear regression controlling for GCS score and the need for intubation. We defined significance at a *p* < 0.05 level. We performed these analyses using SAS 9.4 (Cary, NC, USA).

## Results

### Patient and resuscitation characteristics

Among the 26 patients, most were male (*n* = 23, 88.5%). The average age was 6.5 years (SD 5.2; Table 1). Most patients were injured by a blunt mechanism (*n* = 24, 92.3%), whereas 2 patients (7.7%) had an unknown mechanism of injury but presented with a depressed GCS score with evidence of head trauma (e.g., lacerations, contusions, or abrasions). The median injury severity score (ISS) was 19 (interquartile range [IQR] 6.25–28.5) and median GCS score at presentation was 7.5 (IQR 3.5–10). Twelve patients (46.2%) had a moderate TBI (GCS score 9–12) and 14 (53.8%) had a severe TBI (GCS score 3–8). Seventeen patients (65.4%) required intubation in the pre-hospital setting or during the initial evaluation. Among these patients, 11 (42.3%) were intubated before arrival and 6 (23.1%) after arrival. No patient died in the ED, but 4 patients (15.4%) died before hospital discharge. Most patients arrived following notification of the trauma team (*n* = 22, 84.6%). The remaining 4 patients arrived without notice, requiring the team to assemble after patient arrival. Fourteen patients (53.8%) were triaged to the highest-level attending activation, whereas the other 12 (46.2%) were triaged to lower-level activations. NREs occurred in every case. No association was observed between the number or rate of NREs and whether the activation has at a lower or higher level.

**Table 1.** Summary Statistics

Variable	Resuscitations (*n* = 26)
Age (years), average (SD)	6.5 (5.2)
Female, no. (%)	3 (11.5)
Mechanism, no. (%)	
Blunt	24 (92.3)
Other/Unknown	2 (7.7)
ISS, median (IQR)	19 (6.25–28.5)
Initial GCS score, median (IQR)	7.5 (3.5–10)
TBI severity, no. (%)	
Moderate (GCS score 9–12*)*	12 (46.2)
Severe (GCS score 3–8)	14 (53.8)
Need for intubation, no. (%)	
No intubation	9 (34.6)
Intubation pre-arrival	11 (42.3)
Intubation in ED	6 (23.1)
Trauma activation characteristics, no. (%)	
Arrival without prior notification	4 (15.4)
Night	12 (46.2)
Weekend	9 (34.6)
Mortality, no. (%)	
Died in ED	0 (0)
Hospital death	4 (15.4)

ED, emergency department; GCS, Glasgow Coma Scale; IQR, interquartile range; ISS, Injury Severity Score; SD, standard deviation; TBI, traumatic brain injury.

### Significant physiological disturbances

All but 1 patient (*n* = 25, 96.2%) had episodes of a significant physiological disturbance. Among the significant physiological disturbances associated with poor outcomes following head trauma, respiratory depression was the most common, followed by hypoxia and then hypotension (Table 2). Respiratory depression occurred in 16 patients, with a median percent case duration of 5.7% (IQR 4.1–12.1, range 1.6–24.7). Episodes of hypoxia occurred in 8 patients, with a median percent case duration of 2.2% (IQR 1.8–2.9, range 1.4–29.5). Episodes of hypotension occurred in only 4 patients, with a median percent case duration of 12.7% (IQR 6.5–23.3, range 3.1–40.3). Among the other evaluated significant physiological disturbances, tachypnea was the most frequent, occurring in 18 patients, followed by hypertension (*n* = 17 patients), tachycardia (*n* = 16 patients), and bradycardia (*n* = 7 patients). The tachycardia episodes were associated with the longest median percent duration (29.5%, IQR 12.5–40.1, range 1.8–75).

**Table 2.** Percent of Resuscitation with Significant Physiological Disturbance

Variable	Number of resuscitations with SPD	Percent of cases with SPD, median % (IQR)
Hypotension	4	12.7 (6.5–23.3)
Hypertension	17	29.2 (14.1–57.2)
Bradycardia	7	11.6 (4.1–24.2)
Tachycardia	16	29.5 (12.5–40.1)
Respiratory depression	16	5.7 (4.1–12.1)
Tachypnea	18	26.7 (14.8–32.9)
Hypoxia	8	2.2 (1.8–2.9)

IQR, interquartile range; SPD, significant physiological disturbance.

### NRE rate per resuscitation and domain phase

We identified 604 NREs in the 26 resuscitations (Supplementary Table S1). The median number of NREs per resuscitation was 23 (IQR 17–27.8, range 5–44), and the median rate of NREs per minute over the entire resuscitation was 0.8 (IQR 0.6–0.9, range 0.2–1.6). As resuscitations progressed from the before primary survey to post-secondary survey phases, a greater number of NREs per phase occurred (Table 3). Despite this increase in the absolute number of NREs per phase, the number of NREs per minute decreased as the resuscitation progressed, with a median of 1.2 NREs per minute before the primary survey (IQR 0.7–2, range 0–7.5), 1.1 NREs per minute during the primary survey (IQR 0.6–1.3, range 0–2.4), 0.9 NREs per minute during the secondary survey (IQR 0.6–1, range 0–1.5), and 0.5 NREs per minute during the post-secondary survey phase (IQR 0.3–0.7, range 0.2–0.9). In a multi-variate model, no differences were observed in NRE rate based on intubation status (median 0.6 NREs/min, IQR 0.6–0.9 vs. median 0.8 NREs/min, IQR 0.8–0.9, *p* = 0.21). The rate of NREs was not different between patients who presented with a GCS score <9 compared with those with a higher GCS score (median 0.6, IQR 0.5–0.8 vs. median 0.8, IQR 0.7–0.9, *p* = 0.26). We observed no association between the duration of hypoxia, respiratory depression, and hypotension with the rate of NREs (*p* = 0.58, *p* = 0.89, and *p* = 0.58, respectively).

**Table 3.** Distribution of Non-Routine Events among 26 Resuscitations

	Median (IQR)				
Variable	All phases	Before primary survey	Primary survey	Secondary survey	Post-secondary survey^a^
NRE rate, NRE/min	0.8 (0.6–0.9)	1.2 (0.7–2)	1.1 (0.6–1.3)	0.9 (0.6–1)	0.5 (0.3–0.7)
NRE type	23 (17–27.8)	2 (1–3.8)	4 (2–6.8)	6 (3–8.8)	7 (4–11)
Process events					
Commission	2 (1–4)	0 (0–1)	0 (0–1)	0.5 (0–1)	0 (0–1)
Omission	11 (8–13)	1 (1–2)	1 (0.3–3.8)	3 (2–4.8)	3 (1.3–5)
Selection	4 (2–5)	0 (0–0.8)	1.5 (0–2)	1 (0–1.8)	1 (0–2)
Non-process events	5 (4–6)	0 (0–1)	0 (0–1)	1 (0–2)	2 (2–3)
NREs by team role					
Leadership	3 (1.3–4)	0 (0–0) §	0 (0–1)	0.5 (0–1)	1 (1–2)
Airway	3 (2–5.8)	0 (0–0) §	0 (0–1)	1 (0–2)	1 (0–2)
Surgical surveyor	3 (2–5.5)	0 (0–0.8)	1.5 (0–3)	1 (0–2)	0 (0–1)
Bedside nurse	4 (2.3–6)	0 (0–0) §	0 (0–1)	1 (0–1.8)	1 (1–3)
Neurosurgery	0 (0–1)	0 ǂ	0 (0–0) §	0 (0–0) §	0 (0–1)
EMS technician	1 (1–2)	1 (0–1)	0 (0–0) §	0 (0–0) §	0 (0–0) §
Patient	0 (0–1)	0 ǂ	0 (0–0) §	0 (0–1)	0 (0–0) §
Entire team	1 (0–2)	0 (0–0) §	0 (0–0.8)	0 (0–0.8)	0 (0–0)
Other personnel	0 (0–2)	0 ǂ	0 ǂ	0 (0–0) §	1 (0–2)
Equipment/external	2 (1–3.8)	0 (0–0.8)	0 (0–0.8)	0 (0–1)	1 (0–1)

^a^ Post-secondary phase did not occur in one resuscitation; 0 ǂ indicates no observed events; 0 (0–0) § indicates events occurring with a low frequency with median and IQR of 0.

EMS, emergency medical services; IQR, interquartile range; NRE, non-routine event.

### NRE type and cause

NREs identified as process errors related to team action or inaction were more common than non-process events in every resuscitation (*n* = 465, 77.0%, median 18 NREs, IQR 11.3–23, range 4–38 vs. *n* = 139, 23.0%, median 5 NREs, IQR 4–6, range 0–13; Supplementary Table S1). Among the process errors, errors of omission accounted for more than half of NREs (*n* = 285, 61.3%, median 11 NREs, IQR 8–13, range 3–21) and were observed in all 26 resuscitations. The most common errors of omission were inadequate communication that required clarification (*n* = 104, 36.5%), inadequate use of personal protective equipment, (*n* = 83, 29.1%), and inadequate cervical spine stabilization (*n* = 39, 13.7%). Errors of selection (*n* = 113, 24.3%, median 4 NREs, IQR 2–5, range 0–12) were the next most frequent process errors followed by errors of commission (*n* = 67, 14.4%, median 2 NREs, IQR 1–4, range 0–8). Non-process events (*n* = 139, median 5 NREs, IQR 4–6, range 0–13) unrelated to actions of the assembled team were more common than errors of selection and commission, but less common than errors of omission. The most common non-process events were external distractions to team members, including pages and phone calls (*n* = 48, 34.5%).

Most NREs were attributed to the bedside team (bedside nursing *n* = 122 NREs, 20.2%; airway team *n* = 113 NREs, 18.7%; surgical surveyor *n* = 98 NREs, 16.2%). The most common NRE attributed to bedside nursing and the surgical surveyor was inadequate communication requiring clarification (*n* = 45, 36.9%, *n* = 29, 30%, respectively), and the most common NRE attributed to the airway team was inadequate cervical spine stabilization (*n* = 35, 31%). Bedside nursing and the airway team were the only roles with an attributed NRE in every resuscitation. The leadership team had the next highest number of associated NREs, with 91 identifiable events (15.1%, median 3, IQR 1.3–4, range 0–9), followed by 33 NREs (5.5%, median 0.5, IQR 0–1.8, range 0–10) that could be attributed to the assembled team.

### NRE impact: Time expense and severity

More than half of the identified NREs were associated with a temporal time delay that prevented team members from progressing to their next task (*n* = 384, 63.6%, Table 4, Supplementary Table S1). Momentary delays causing NREs were the most common and were identified in every resuscitation (*n* = 352, median 13 NREs/resuscitation, IQR 8–17.8, range 2–29). The most frequently identified momentary delay causing NRE was inadequate communication requiring clarification (*n* = 104, 29.5%). Moderate delays occurred in 19 resuscitations (*n* = 32, median 1 NRE/resuscitation, IQR 1–2, range 1–5) and were most frequently associated with difficulty obtaining intravenous access (*n* = 7, 21.9%).

**Table 4.** Impact of Non-Routine Events

Type of impact	Resuscitations (*n* = 26)
Time expense, median (IQR)	
No delay	8 (6–10)
Momentary delay	13 (8–17.8)
Moderate delay	1 (0.3–1)
Severity, median (IQR)	
Minor	17.5 (15–21)
Major	5 (3–7)

IQR, interquartile range.

We found no association between the duration of hypotensive, desaturation, and respiratory depression events, and the rate of NREs contributing to a momentary delay (*p* = 0.58, *p* = 0.89, *p* = 0.58, respectively). Desaturation and respiratory depression events were associated with a greater rate of moderate NREs (*p* = 0.008 and *p* < 0.001, respectively), whereas hypotension events were associated with a lower rate of moderate NREs (*p* = 0.005). NREs that were minor in severity were more frequent than those that could lead to major harm (*n* = 467, 77.3%, median 17.5 NREs/resuscitation, IQR 15–21, range 5–37 vs. *n* = 137, 22.7%, median 5 NREs/resuscitation, IQR 3–7, range 0–13). Minor NREs were most frequently related to inadequate communication requiring clarification (*n* = 104, 22.3%). The most common major NRE was inadequate cervical spine stabilization (*n* = 39, 28.5%). We found no association between hypotensive, desaturation, and respiratory depression events and the rate of major NREs per resuscitation (*p* = 0.57, *p* = 0.30, *p* = 0.39, respectively).

## Discussion

During the initial resuscitation of children with moderate to severe head trauma, we observed that NREs were frequent. We also found that vital sign derangements occurred in almost every resuscitation. Previous work identifying NREs in the operating room showed that cases containing NREs have more vital sign derangements than those without NREs.^27^ In contrast, our study did not find an association between the overall NRE rate and vital sign abnormalities, consistent with our prior work in this setting.^21^ Although we observed no association between the overall NRE rate and vital sign abnormalities, the rate of NREs causing moderate delays in care were associated with respiratory depression and hypoxia events. We also observed no association between GCS score severity, need for intubation, and the rate of NREs.^21^

Vital sign monitoring early after injury is needed for the identification of derangements in blood pressure and oxygen saturation. Maintenance of blood pressure goals supports cerebral perfusion pressure and prevents secondary brain injury.^16^ Hypotension in the early resuscitation phase following TBI is associated with worsened Glasgow Outcome Scale (GOS) scores at discharge and higher mortality.^28–30^ If unaddressed, hypotension increases the likelihood of disability and in-hospital mortality by greater than three-fold.^5,17^ Hypotension was infrequently observed in this current study. Although a poor prognostic indicator, an elevated age-adjusted shock index (e.g., tachycardia, hypotension) is uncommonly observed in patients with TBI.^31^

Unlike hypotension, hypoxia was a commonly observed vital sign derangement. Among children with severe TBI, early hypoxia is common, often requiring the rapid administration of oxygen by a non-invasive (e.g., non-rebreather) or invasive approach (e.g., intubation).^16^ Unmanaged hypotension and hypoxia have a synergistic effect on mortality, leading to a three-fold increase compared with either derangement alone.^5^ In our study, hypoxia and respiratory depression were more frequent than episodes of hypotension, and were associated with a greater rate of NREs contributing to a moderate delay in patient care. The higher frequency of NREs suggests that hypotension may be perceived as a more immediate problem that is addressed more quickly than hypoxia or respiratory depression.^32^

During the initial resuscitation of children with TBI, adherence to ATLS and PALS guidelines have been linked to improved outcomes, including lower long-term functional impairment and mortality.^33,34^ Deviations from the established protocols are often errors of omission in which key aspects of the resuscitation are skipped or delayed.^21,22,35^ Other studies in the setting of trauma resuscitations have found errors of omission to be the most common error type, which are often more severe and associated with more adverse patient events.^22^ In our study, errors of omission were more common that other process errors and were more frequent than non-process events.

Studies using video review of trauma resuscitations have found that more than 10 deviations from ATLS guidelines occur per resuscitation.^21,22,36^ Greater than 40% of preventable or potentially preventable deaths following injury can be attributed to errors occurring during the initial phase of resuscitation, which increases the importance of guideline adherence during the early post-injury phase.^9,37–39^ Protocol adherence may be increased through the use of clinical decision support (e.g., checklists, vital sign alerts) that can guide the resuscitation and ensure efficient progress forward in this phase.^40,41^ Despite the association between guideline adherence and improved GOS scores, variability is frequently observed during resuscitation after TBI.^8,16,28,29^ Variability in guideline adherence has been attributed to perceptions that the guidelines do not apply to the individual patient or to failure of guideline implementation by the team leader.^33^

In our study, we observed that errors of omission were common. Among the identified errors of omission, failure to maintain cervical spine immobilization was the most frequent NRE associated with the potential for harm. Errors in cervical spine management are common during trauma resuscitations, occurring most often during the primary and secondary surveys.^37^ Despite the rarity of cervical spine injuries in the pediatric population, cervical spine injuries are highly associated with TBI.^42,43^ This association requires the maintenance of cervical spine stability in patients with moderate to severe TBI.^44–48^ Cervical spine immobilization is a responsibility of an individual in close proximity to the patient's airway. In some settings, a respiratory therapist may be responsible for managing both the airway and maintaining cervical spine alignment. Having a provider responsible for simultaneous tasks may explain the frequency of lapses in cervical spine stabilization.^37^ In our study, a lapse in cervical spine stabilization occurred more than once per resuscitation. Both the rate of cervical-spine-related near-miss events and the rate of lapses in cervical spine immobilization causing patient harm can be decreased by adherence to protocols for cervical spine stability.

We also frequently observed NREs related to inadequate communication between team members. Communication during trauma resuscitations is often inadequate or unclear, and this can lead to delays in interventions and the transmission of inaccurate information during the resuscitation.^49^ We found that bedside team members often needed to repeat exam findings to convey accurate information to the team leadership. Repetition and clarification of information required the bedside team members to stop their current tasks, leading to brief delays in patient care. Closed-loop communication is a method that may reduce delays, increase task completion efficiency, and decrease the uncertainty associated with reporting important exam findings that are not initially acknowledged by the team leadership.^50–52^

This study has several limitations. First, NRE identification requires a subjective assessment. We limited this factor by using two reviewers for each case with initial training and assessment of coding performance before initiation of the study. Second, video review is subject to limitations in image and audio quality. The cameras used to record trauma resuscitations provide a fixed view of the trauma bay and are unable to capture activities occurring outside this field. In our trauma bay, we use two cameras with different views of the patient treatment stretcher to optimize the field of view. Finally, this study was performed at a single institution. Confirmation of our findings will require verification at other sites.

NREs are common during resuscitations of children with TBIs. NREs associated with a moderate delay in care are associated with increased incidence of hypoxia and respiratory depression, factors associated with poor outcomes after TBI. The use of methods such as closed-loop communication and clear assignment of roles and responsibilities are strategies that may mitigate the occurrence of NREs and increase the efficacy of task performance. This study identifies the most common NREs associated with resuscitations for pediatric head trauma. Our results can serve as a guide for developing interventions in the early phase of care that reduce morbidity and mortality associated with pediatric TBI.

## Supplementary Material

Supplemental data

## Abbreviations Used

ATLS: Advanced Trauma Life Support
ED: emergency department
EMS: emergency medical services
GCS: Glasgow Coma Scale
GOS: Glasgow Outcome Scale
IQR: interquartile range
ISS: injury severity score
NRE: non-routine event
PALS: Pediatric Advanced Life Support
SD: standard deviation
SPD: significant physiological disturbance
TBI: traumatic brain injury
