# Supplementary material for: Assessment of Non-Routine Events and Significant Physiological Disturbances during Emergency Department Evaluation after Pediatric Head Trauma
Source: Neurotrauma Rep. 2021 Jan 29;2(1):39–47. doi: 10.1089/neur.2020.0043 (PMC7962792; doi:10.1089/neur.2020.0043)
Supplement: Supplemental data [file Supp_TablseS1.docx]

| **Appendix.** Non-routine events occurring during 26 pediatric trauma resuscitations for traumatic brain injury* | | | | | | |  | | |  |  |
| --- | --- | --- | --- | --- | --- | --- | --- | --- | --- | --- | --- |
| **Non-routine event** | | | **Total NREs** | **Associated time expense** | | | | **Potential for Harm** | | | |
|  |  |  |  | No delay | Momentary delay | Moderate delay | | Minor | Major | | |
| **Errors of Commission:** events in which an incorrect action was taken to address a goal | | | | | | | | | | | |
|  | **Commission Irrelevant**: if conditions for establishing the goal are known to be false | | | | | | | | | | |
|  |  | *Non-critical task for patient care-* team member performs task unrelated to patient care Example: placing a pillow under the patient’s head for comfort | **1** | 0 | 1 | 0 | | 1 | 0 | | |
|  |  | *Non-routine request to remove something from patient*- request made that a team member remove an item from the patient not usually necessary for removal  Example: removal of earrings | **0** | 0 | 0 | 0 | | 0 | 0 | | |
|  |  | *Request for a non-standard object-* request by team member for something unusual in the setting of trauma resuscitation Example: requesting a paperclip for an X-ray marker | **0** | 0 | 0 | 0 | | 0 | 0 | | |
|  |  | *Unusual request (situational)*- request by team member for a situational change in the environment to facilitate care Example: turning the lights off for a pupillary exam | **1** | 0 | 1 | 0 | | 1 | 0 | | |
|  | **Commission Premature:** if some, but not all, conditions for establishing the goal are true | | | | | | | | | | |
|  |  | *Failure to appropriately notify team of actions-* initiation of team actions without informing the whole team Example: initiating log roll | **2** | 0 | 2 | 0 | | 2 | 0 | | |
|  |  | *Patient still connected to equipment at time of movement-­* patient still attached to non-mobile equipment when the bed begins to leave the room | **2** | 0 | 2 | 0 | | 2 | 0 | | |
|  |  | *Premature removal of supplemental oxygen-* oxygen delivery device is removed from patient even though it is still required | **1** | 1 | 0 | 0 | | 0 | 1 | | |
|  | **Commission Prohibited:** if action is contraindicated for medical or logistical reasons | | | | | | | | | | |
|  |  | *Dropped equipment-* dropped equipment or care items have to be picked up to continue care | **6** | 0 | 6 | 0 | | 6 | 0 | | |
|  |  | *Glasgow coma score (GCS)* inaccurate*-* incorrect GCS calculation is verbalized and must be revised | **3** | 1 | 2 | 0 | | 3 | 0 | | |
|  |  | *Inaccurate interpretation-* a concerning exam finding is interpreted as normal  Example: Reporting a GCS of 6 without a discussion about intubation following | **1** | 0 | 0 | 1 | | 0 | 1 | | |
|  |  | *Inadequate bagging/ventilation (too fast)-* hyperventilation of the patient | **2** | 1 | 1 | 0 | | 0 | 2 | | |
|  |  | *Inadequate bagging/ventilation (too slow)-* hypoventilation of the patient | **4** | 2 | 2 | 0 | | 0 | 4 | | |
|  |  | *Inadequate chest compressions (too fast)-* speed of chest compressions is too fast | **0** | 0 | 0 | 0 | | 0 | 0 | | |
|  |  | *Inadequate chest compressions (too slow)-* speed of chest compressions is too slow | **0** | 0 | 0 | 0 | | 0 | 0 | | |
|  |  | *Incorrect airway assessment-* incorrect information or technique used to assess the airway | **0** | 0 | 0 | 0 | | 0 | 0 | | |
|  |  | *Incorrect otoscope technique*- evaluating ear from the opposite side of the bed | **4** | 4 | 0 | 0 | | 4 | 0 | | |
|  |  | *Mask size mismatch*- inappropriately sized oxygen mask resulting in inadequate ventilation | **0** | 0 | 0 | 0 | | 0 | 0 | | |
|  |  | *Noise level (room)*- excessive level of noise in the room related to conversation requiring a team member to request silence | **15** | 1 | 14 | 0 | | 15 | 0 | | |
|  |  | *Other unspecified technique failure*- care actions not specified elsewhere that are done incorrectly Example: blood pressure cuff placed upside down | **1** | 0 | 1 | 0 | | 1 | 0 | | |
|  |  | *Poor quality chest compressions*- chest compressions that are not deep enough or done with incorrect technique but at the correct speed | **0** | 0 | 0 | 0 | | 0 | 0 | | |
|  |  | *Problem securing tube*- ineffective method for securing endotracheal tube leading to delay Example: taping fingers to tube | **4** | 0 | 0 | 4 | | 0 | 4 | | |
|  |  | *Tube reposition*- advancement or pulling back on endotracheal tube after initial placement | **4** | 0 | 2 | 2 | | 0 | 4 | | |
|  |  | *Unable to find equipment-* team member looks for piece of medical equipment but cannot find item | **2** | 0 | 2 | 0 | | 1 | 1 | | |
|  |  | *Unable to find gloves-* team member looks for gloves but cannot find them | **0** | 0 | 0 | 0 | | 0 | 0 | | |
|  |  | *Unable to pass tube-* anesthesiologist unable to pass endotracheal tube despite adequate visualization | **1** | 0 | 1 | 0 | | 0 | 1 | | |
|  |  | *Unsafe environment for team members (sharps)-* needles or other sharp objects are incorrectly disposed of | **1** | 0 | 1 | 0 | | 1 | 0 | | |
|  |  | *Unsafe environment for team members (stretcher movement)-* moving stretcher is pushed into wall or equipment | **7** | 7 | 0 | 0 | | 7 | 0 | | |
|  |  | *Unsafe environment for team members (stretcher use)-* team member rides on stretcher with patient | **0** | 0 | 0 | 0 | | 0 | 0 | | |
|  |  | *Unsafe environment for team members (tripping)-* team member trips over cords or equipment | **0** | 0 | 0 | 0 | | 0 | 0 | | |
|  |  | *Ventilator disconnected-* accidental or unplanned disconnection of ventilator | **3** | 0 | 3 | 0 | | 0 | 3 | | |
|  |  | *Wrong blade-* incorrect size or type of laryngoscope blade provided | **0** | 0 | 0 | 0 | | 0 | 0 | | |
|  |  | *Wrong dose-*incorrect dose of appropriate medication provided | **0** | 0 | 0 | 0 | | 0 | 0 | | |
|  |  | *Wrong medication-* incorrect medication drawn but not administered | **0** | 0 | 0 | 0 | | 0 | 0 | | |
|  |  | *Wrong size cervical collar-* cervical collar too small or large and is changed due to poor fit | **2** | 0 | 2 | 0 | | 2 | 0 | | |
|  |  | *Wrong size tube-* change in endotracheal tube following initial attempt at intubation with a tube that is too small or big | **0** | 0 | 0 | 0 | | 0 | 0 | | |
| **Total No. of Errors of Commission (% of all NREs)** | | | **67 (11.1)** | **17 (2.8)** | **43  (7.1)** | **7 (1.2)** | | **46 (7.6)** | **21 (3.5)** | | |
| **Errors of Omission:** events that involved no or inadequate action to address a goal despite action being required | | | | | | | | | | |  |
|  | **Omission Partial:** errors of omission where some actions have been done to address the goal | | | | | | | | | | |
|  |  | *Inaccurate communication*- good quality communication occurs but conveys inaccurate information  Example: stating “GCS is 10” when GCS is 8 | **15** | 2 | 13 | 0 | | 12 | 3 | | |
|  |  | *Inadequate communication (including clarification)-* communication occurs but is partial or confusing and requires clarification  Example: Initial statement that right pupil is dilated corrected to left pupil is dilated | **104** | 0 | 104 | 0 | | 104 | 0 | | |
|  |  | *Inadequate sedation-* patient not adequately sedated and reacts to the endotracheal tube requiring more sedation to prevent unintended endotracheal tube dislodgement | **10** | 3 | 7 | 0 | | 0 | 10 | | |
|  |  | *Inadequate voice projection*- communication occurs but is unable to be heard by the nurse documenter Example: nurse documenter asks surgical surveyor to repeat information | **16** | 0 | 16 | 0 | | 16 | 0 | | |
|  |  | *Insufficient clothing or pre-hospital equipment removal-* lack of complete clothing removal interferes with examination | **1** | 1 | 0 | 0 | | 1 | 0 | | |
|  |  | *Lines tangled-* tangled lines or tubes inhibit care | **3** | 0 | 3 | 0 | | 3 | 0 | | |
|  | **Omission Total:** errors of omission where nothing has been done to address the goal | | | | | | | | | | |
|  |  | *Airway not assessed-* no verbal report or apparent assessment of the airway | **0** | 0 | 0 | 0 | | 0 | 0 | | |
|  |  | *Failure to ever upgrade to higher level trauma at decision to intubate-* appropriate upgrade in trauma activation level not made | **0** | 0 | 0 | 0 | | 0 | 0 | | |
|  |  | *GCS not obtained*- GCS not obtained during primary survey | **1** | 1 | 0 | 0 | | 0 | 1 | | |
|  |  | *Inability to draw blood-* failure to draw blood from patient due to the skills of the team member attempting the blood draw | **3** | 0 | 0 | 3 | | 0 | 3 | | |
|  |  | *Inability to place intraosseous line-* failure to obtain intraosseous line due to skills of team member attempting placement, line not in place when needed | **0** | 0 | 0 | 0 | | 0 | 0 | | |
|  |  | *Inability to place intravenous catheter*- failure to obtain intravenous access due to skills of team member attempting placement, line not in place when needed | **8** | 0 | 1 | 7 | | 0 | 8 | | |
|  |  | *Inability to place orogastric or nasogastric tube -* inability to place an orogastric or nasogastric tube despite appropriate attempt | **0** | 0 | 0 | 0 | | 0 | 0 | | |
|  |  | *Inadequate personal protective equipment-* examination without gloves or doing a procedure without a gown and gloves | **83** | 83 | 0 | 0 | | 83 | 0 | | |
|  |  | *Incorrect technique (pulse checks)*- central pulses not palpated during cardiopulmonary resuscitation | **0** | 0 | 0 | 0 | | 0 | 0 | | |
|  |  | *No attempts at clothing removal-* patient clothing not removed at all during the resuscitation | **0** | 0 | 0 | 0 | | 0 | 0 | | |
|  |  | *No cervical spine stabilization*- failure to hold in-line cervical spine stabilization in a patient without a cervical collar or prior clearance | **39** | 38 | 1 | 0 | | 0 | 39 | | |
|  |  | *No chest compressions when indicated*- chest compressions not done with a non-perfusable rhythm | **0** | 0 | 0 | 0 | | 0 | 0 | | |
|  |  | *Not obtaining temperature*- temperature not obtained during the resuscitation | **0** | 0 | 0 | 0 | | 0 | 0 | | |
|  |  | *Pupils not checked*- pupillary exam not done during primary survey | **0** | 0 | 0 | 0 | | 0 | 0 | | |
|  |  | *X-ray equipment left behind*- equipment left behind after X-ray completion | **2** | 2 | 0 | 0 | | 2 | 0 | | |
| \| **Total No. of Errors of Omission (% of all NREs)** \|  \|  \|  \|  \|  \| \| --- \| --- \| --- \| --- \| --- \| --- \| | | | **285 (47.2)** | **130 (21.5)** | **145  (24)** | **10  (1.7)** | | **221 (36.6)** | **64**  **(10.6)** | | |
| **Selection Errors:** suboptimal choices among potential options | | | | | | | | | | | |
|  | **Selection Precedence:** certain procedures should always be done in a given order because performance of one affects the other | | | | | | | | | | |
|  |  | *Anesthesia not ready*- anesthesiologist present but not ready to begin the intubation | **0** | 0 | 0 | 0 | | 0 | 0 | | |
|  |  | *Bair Hugger™ (not on or disconnected)*- external warming equipment not turned on or gets disconnected | **14** | 10 | 4 | 0 | | 14 | 0 | | |
|  |  | *Cell phone light use*- cell phone light used to check pupils even though otoscope is available | **10** | 10 | 0 | 0 | | 10 | 0 | | |
|  |  | *Delay in 3% saline administration*- need for 3% saline is recognized but administration is delayed | **0** | 0 | 0 | 0 | | 0 | 0 | | |
|  |  | *Delay in airway assessment*- airway evaluation late or has to be requested | **1** | 0 | 1 | 0 | | 0 | 1 | | |
|  |  | *Delay in applying oxygen*- oxygen not applied on arrival | **2** | 0 | 2 | 0 | | 0 | 2 | | |
|  |  | *Delay in cervical collar placement*- delayed cervical collar placement when indicated | **4** | 0 | 4 | 0 | | 0 | 4 | | |
|  |  | *Delay in decision to intubate*- obvious need for intubation recognized but delayed | **0** | 0 | 0 | 0 | | 0 | 0 | | |
|  |  | *Delay in intravenous fluid bolus administration-* need for fluid bolus recognized but administration is delayed | **0** | 0 | 0 | 0 | | 0 | 0 | | |
|  |  | *Delay in mannitol administration-* need for mannitol recognized but administration is delayed | **0** | 0 | 0 | 0 | | 0 | 0 | | |
|  |  | *Delay in medication administration-* administration of prepared medication is late or must be requested | **2** | 0 | 2 | 0 | | 0 | 2 | | |
|  |  | *Delay in obtaining GCS*- GCS requested but should have already been obtained | **1** | 0 | 1 | 0 | | 0 | 1 | | |
|  |  | *Delay in obtaining intravenous access*- intravenous access not obtained during the primary or early secondary survey | **0** | 0 | 0 | 0 | | 0 | 0 | | |
|  |  | *Delay in obtaining temperature-* temperature requested but should have already been obtained | **3** | 1 | 2 | 0 | | 0 | 3 | | |
|  |  | *Delayed clothing removal*- clothing not removed during primary survey | **2** | 1 | 1 | 0 | | 2 | 0 | | |
|  |  | *Delayed trauma activation upgrade to highest level at decision to intubate*- appropriate but delayed upgrade in trauma level (greater than five minutes after the decision to intubate) | **0** | 0 | 0 | 0 | | 0 | 0 | | |
|  |  | *Deviation from ATLS or other protocol*- leadership must direct team member to return to or complete a required task | **28** | 0 | 25 | 3 | | 24 | 4 | | |
|  |  | *End-tidal CO_2_ problem (not set up)*- problem related to end-tidal CO_2_ device that is not an equipment malfunction | **7** | 0 | 7 | 0 | | 0 | 7 | | |
|  |  | *Intravenous catheter not in place when needed-* intravenous catheter not in place when medications administration is needed | **1** | 0 | 0 | 1 | | 0 | 1 | | |
|  |  | *Intravenous flush not provided-* saline flush not provided when needed | **1** | 0 | 1 | 0 | | 1 | 0 | | |
|  |  | *Laryngoscope blade not prepared*- appropriate laryngoscope blade not ready for use | **0** | 0 | 0 | 0 | | 0 | 0 | | |
|  |  | *Medication not ready for administration*- a medication has not been drawn up when needed | **4** | 0 | 2 | 2 | | 0 | 4 | | |
|  |  | *No or delayed chest auscultation (intubation)*- failure to listen to breath sounds immediately after intubation | **1** | 0 | 1 | 0 | | 0 | 1 | | |
|  |  | *No stethoscope available for auscultation-* team member needs to borrow a stethoscope for assessment | **2** | 0 | 2 | 0 | | 2 | 0 | | |
|  |  | *Other delay in appropriate action-* appropriate actions done but delayed- delay is acknowledged and action must be requested  Example delay in applying a dressing to a bleeding head wound | **3** | 0 | 3 | 0 | | 1 | 2 | | |
|  |  | *Other unspecified personnel failure*- unspecified task not completed or completed in a delayed fashion due to team member actions Example: disagreement between team members delaying intubation | **0** | 0 | 0 | 0 | | 0 | 0 | | |
|  |  | *Poor patient positioning for intubation-* patient has to be moved or repositioned on the stretcher for intubation | **3** | 0 | 3 | 0 | | 3 | 0 | | |
|  |  | *Poor positioning (other)-* patient adjusted on the stretcher for safe transport out of the room | **11** | 1 | 10 | 0 | | 11 | 0 | | |
|  |  | *Poor positioning for X-ray*- patient repositioned on the stretcher for X-ray (beyond normal repositioning) | **9** | 0 | 9 | 0 | | 9 | 0 | | |
|  |  | *Suction not available-* suction not set up when needed or requested | **0** | 0 | 0 | 0 | | 0 | 0 | | |
|  |  | *Team not prepared for backboard removal-* failure to remove the board or the board is caught on equipment during log roll | **0** | 0 | 0 | 0 | | 0 | 0 | | |
|  | **Selection Precondition**: a precondition for a procedure has not been satisfied | | | | | | | | | | |
|  |  | *Delay in chest auscultation-*  delay in chest auscultation before intubation | **0** | 0 | 0 | 0 | | 0 | 0 | | |
|  |  | *Delay in circulatory assessment-* appropriate technique used but pulses checked later than expected in the primary survey or on repeat examination | **4** | 0 | 3 | 1 | | 0 | 4 | | |
|  |  | *Laryngoscope blade is requested-* appropriate laryngoscope blade needs to be requested because it is not immediately available for the anesthesiologist | **0** | 0 | 0 | 0 | | 0 | 0 | | |
| \| **Total No. of Selection Errors (% of all NREs)** \|  \|  \|  \|  \|  \| \| --- \| --- \| --- \| --- \| --- \| --- \| | | | **113 (18.7)** | **23 (3.8)** | **83 (13.7)** | **7 (1.2)** | | **77 (12.7)** | **36 (6)** | | |
| **Non-Process Events:** events that exist external to the actions of the assembled team | | | | | | | | | | | |
|  | **Non-Process Aberrant Personnel:** atypical person filling a particular role or no person present to fill a particular role^+^ | | | | | | | | | | |
|  |  | *Anesthesiologist late/absent-* anesthesiologist not present when patient requires intubation but arrives eventually or is absent from resuscitation | **4** | 2 | 0 | 2 | | 0 | 4 | | |
|  |  | *Lead nurse late/absent-* lead nurse/nurse documenter not present when patient arrives but arrives eventually or is absent from resuscitation | **3** | 2 | 1 | 0 | | 3 | 0 | | |
|  |  | *Neurosurgeon not present within 30 minutes of patient arrival time-* neurosurgeon arrives outside the 30-minute window or does not arrive at all | **5** | 5 | 0 | 0 | | 5 | 0 | | |
|  |  | *Respiratory therapist late/absent- respiratory therapist* not present when patient arrives but arrives eventually or is absent from resuscitation | **1** | 1 | 0 | 0 | | 1 | 0 | | |
|  |  | *Surgical coordinator late/absent*- surgical coordinator not present when patient arrives but arrives eventually or is absent from resuscitation | **6** | 6 | 0 | 0 | | 6 | 0 | | |
|  |  | *Surgical surveyor late/absent*- surgical junior not present when patient arrives but arrives eventually or is absent from resuscitation | **5** | 5 | 0 | 0 | | 5 | 0 | | |
|  |  | *Trauma attending not present within 15-minutes of activation upgrade-* surgical attending arrives outside the 15-minute window | **4** | 4 | 0 | 0 | | 4 | 0 | | |
|  | **Non-Process Equipment Malfunction:** equipment is not working properly or needs adjustment | | | | | | | | | | |
|  |  | *Bair Hugger™ malfunction-* external warming device stops working | **0** | 0 | 0 | 0 | | 0 | 0 | | |
|  |  | *End-tidal CO_2_ problem (not working)*- end-tidal CO_2_ device does not work despite appropriate setup | **3** | 0 | 3 | 0 | | 0 | 3 | | |
|  |  | *Hospital obtained access failure*- intravenous or intraosseous catheter placed in the emergency department stops working | **1** | 1 | 0 | 0 | | 0 | 1 | | |
|  |  | *Other unspecified equipment failure*- failure of equipment that is not specified elsewhere Example: excessive static from Doppler machine | **6** | 0 | 6 | 0 | | 6 | 0 | | |
|  |  | *Otoscope equipment failure*- otoscope light does not work | **1** | 0 | 1 | 0 | | 1 | 0 | | |
|  |  | *Poor lighting-* overhead lighting is not adequate for visualization | **0** | 0 | 0 | 0 | | 0 | 0 | | |
|  |  | *Stretcher movement- the* stretcher moves because the brakes are not working | **0** | 0 | 0 | 0 | | 0 | 0 | | |
|  |  | *Thermometer malfunction-* thermometer not working and unable to obtain temperature | **10** | 0 | 10 | 0 | | 10 | 0 | | |
|  |  | *Transfer of patient difficult-* difficulty moving patient from pre-hospital stretcher  Example: straps get stuck and patient is almost dropped | **4** | 0 | 2 | 2 | | 3 | 1 | | |
|  |  | *Travel monitor failure-* travel monitor does not work | **0** | 0 | 0 | 0 | | 0 | 0 | | |
|  |  | *X-ray process failure*- X-ray equipment malfunctions and cannot capture image | **0** | 0 | 0 | 0 | | 0 | 0 | | |
|  | **Non-Process External:** NRE comes from outside the code team or originates before patient arrival | | | | | | | | | | |
|  |  | *Activation without pre-arrival notification*- overhead page occurs at the time of patient arrival. There is no prior notification of the trauma team | **4** | 4 | 0 | 0 | | 4 | 0 | | |
|  |  | *Bed position*- stretcher in the wrong position upon patient arrival | **1** | 0 | 1 | 0 | | 1 | 0 | | |
|  |  | *Change to trauma activation*- change from medical activation or change from a non-activation status to a trauma activation | **1** | 1 | 0 | 0 | | 1 | 0 | | |
|  |  | *Failure of access placed in field-* intravenous or intraosseous catheter placed in the pre-hospital setting no longer works | **4** | 2 | 1 | 1 | | 0 | 4 | | |
|  |  | *Intensive care unit bed availability*- intensive care unit team not ready to accept the patient at the time of completion of the resuscitation | **1** | 0 | 0 | 1 | | 1 | 0 | | |
|  |  | *Operating room availability*-surgical team is not ready to accept the patient at the time of completion of the resuscitation | **1** | 0 | 0 | 1 | | 0 | 1 | | |
|  |  | *Over-activation*- inappropriately high level of activation with or without downgrade | **0** | 0 | 0 | 0 | | 0 | 0 | | |
|  |  | *Second simultaneous trauma or medical activation-* another trauma or medical activation occurs at the same time | **3** | 2 | 1 | 0 | | 3 | 0 | | |
|  |  | *Too many people/crowd control*- excessive number of non-involved people present in the room | **3** | 0 | 3 | 0 | | 3 | 0 | | |
|  |  | *Unpredicted events unrelated to patient care*- unclassified external distractions  Example: yelling in the hallway | **0** | 0 | 0 | 0 | | 0 | 0 | | |
|  | **Non-Process Family Action:** NRE involves actions performed by the patient’s family | | | | | | | | | | |
|  |  | *Parental participation in care*- parents provide care to patient or are at bedside impeding workflow | **12** | 12 | 0 | 0 | | 12 | 0 | | |
|  | | | | | | | | | | | |
|  | **Non-Process Interruption:** NRE is a non-process event that may stop task flow | | | | | | | | | | |
|  |  | *Lights*- room lights not on when the patient arrives | **0** | 0 | 0 | 0 | | 0 | 0 | | |
|  |  | *Phone calls and pages*- texting, phone calls, or pages distract the bedside team members and create a delay in care | **48** | 1 | 47 | 0 | | 48 | 0 | | |
|  | **Non-Process Patient Condition:** NRE involves the patient’s requests or actions | | | | | | | | | | |
|  |  | *Combative patient*- patient is uncooperative with the exam or is dangerous to self and staff | **1** | 0 | 0 | 1 | | 0 | 1 | | |
|  |  | *Patient requests to change position-* patient requests to change position due to their injuries | **0** | 0 | 0 | 0 | | 0 | 0 | | |
|  |  | *Upgrade to highest level activation*- upgrade to the highest level activation because of clinical decline | **2** | 2 | 0 | 0 | | 2 | 0 | | |
|  | **Non-Process Unpreparedness:** team does not have appropriate equipment to complete tasks or complete tasks correctly | | | | | | | | | | |
|  |  | *Equipment unavailable*- requested equipment unable to be provided | **4** | 0 | 4 | 0 | | 3 | 1 | | |
|  |  | *Intravenous equipment not available*- needed supplies are not stocked in the trauma bay for intravenous catheter placement | **1** | 0 | 1 | 0 | | 1 | 0 | | |
| \| **Total No. of Non-Process Events (% of all NREs)** \|  \|  \|  \|  \|  \| \| --- \| --- \| --- \| --- \| --- \| --- \| | | | **139 (23.0)** | **50 (8.3)** | **81 (13.4)** | **8 (1.3)** | | **123 (20.4)** | **16 (2.6)** | | |

* Examples are provided when a single specific NRE can be applied to several related events

⁺ Late arrival were coded in the phase of the individual’s arrival arrive and absence in the phase when arrival of the role was required
